# Subversion of inflammasome activation and pyroptosis by pathogenic bacteria

**DOI:** 10.3389/fcimb.2013.00076

**Published:** 2013-11-26

**Authors:** Larissa D. Cunha, Dario S. Zamboni

**Affiliations:** Department of Cell Biology, Ribeirão Preto Medical School, University of São Paulo (FMRP/USP)Ribeirão Preto, Brazil

**Keywords:** inflammasome inhibition, pyroptosis, infection control, subversion strategies

## Abstract

Activation of the inflammasome occurs in response to a notably high number of pathogenic microbes and is a broad innate immune response that effectively contributes to restriction of pathogen replication and generation of adaptive immunity. Activation of these platforms leads to caspase-1- and/or caspase-11-dependent secretion of proteins, including cytokines, and induction of a specific form of cell death called pyroptosis, which directly or indirectly contribute for restriction of pathogen replication. Not surprisingly, bona fide intracellular pathogens developed strategies for manipulation of cell death to guarantee intracellular replication. In this sense, the remarkable advances in the knowledge of the inflammasome field have been accompanied by several reports characterizing the inhibition of this platform by several pathogenic bacteria. Herein, we review some processes used by pathogenic bacteria, including *Yersinia* spp., *Pseudomonas aeruginosa, Vibrio parahaemolyticus, Chlamydia trachomatis, Francisella tularensis, Shigella flexneri, Legionella pneumophila*, and *Coxiella burnetii* to evade the activation of the inflammasome and the induction of pyroptosis.

## Introduction

Host pattern recognition receptors (PRRs) are capable of sensing conserved microbial molecules, referred as Pathogen-Associated Molecular Patterns (PAMPs) as well as cellular disturbances, referred as Damage-Associated Molecular Patterns (DAMPs). PRR activation usually leads to induction of pro-inflammatory signaling networks that facilitate direct elimination of the pathogens but also to alert the immune system. Consequently, successful replication of an intracellular infectious agent relies not only on the arsenal of virulence factors that modulate host cell functions to establish a replicative niche, but also in the development of efficient subversion strategies to evade host recognition and bypass the host mechanisms related to restriction of pathogen replication.

Induction of cell death pathways is a conserved host response to infection. However, different subtypes of cell death can be triggered and they will vary according to many factors, e.g., the type of infected cell and the surrounding environment, the infectious agent and the infection dosage. Interestingly, the same mechanism of cell death can elicit either an immunogenic or a tolerogenic (“silent”) effect upon the immune system, however, the factors controlling such plasticity remain elusive (Green et al., 2009). Apoptosis, autophagy, and necrosis are still considered the main types of cell death, but several other subtypes can be distinguished based mostly on biochemical and functional criteria (Galluzzi et al., 2012).

**Figure 1:**
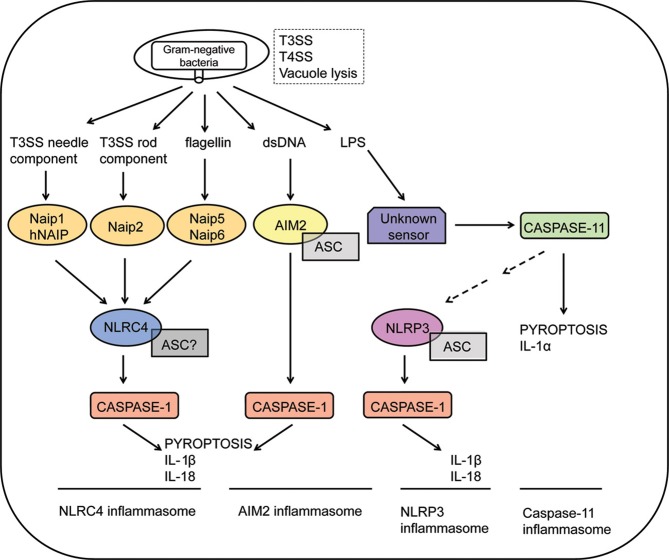
**Inflammasome activation in response to Gram-negative bacteria**. Intracellular sensing of Gram-negative bacteria that violate cytosolic compartments by expressing type III or type IV secretion systems (T3SS and T4SS, respectively) or by inducing vacuolar lysis. NLRC4 inflammasomes are activated in response to recognition of bacterial flagellin by Naip5, T3SS needle proteins by murine Naip1 or by human NAIP (hNAIP), or T3SS rod proteins by Naip2. Activation of the NLRC4 inflammasomes culminates in caspase-1 activation, leading to IL-1β/IL-18 secretion and pyroptosis. The requirement of ASC to the assembly of the NLRC4 inflammasomes is still controversial. Recognition of cytosolic DNA by AIM2 leads to formation of a AIM2/ASC/Caspase-1 multimeric complex known as the AIM2 inflammasome. Cytosolic LPS of Gram-negative bacteria are recognized by an unknown receptor, triggering activation of caspase-11. This process is independent on ASC, NLRP3 and caspase-1, inducing pyroptosis and secretion of IL-1α. Non-canonical inflammasome activation mediated by caspase-11 also regulates NLRP3 activation by unclear mechanisms. Finally, formation of the NLRP3/ASC/caspase-1 complex leads to the secretion of inflammatory cytokines such as IL-1β and IL-18.

Of note, activation of intracellular PRRs belonging to the family of Nod-like receptors (NLRs) or the nucleic acid receptors AIM2 and IFI16 (members of the PYHIN family) trigger a specific type of potentially pro-inflammatory, caspase-1-dependent cell death program known as pyroptosis (Cookson and Brennan, 2001; Lamkanfi and Dixit, 2012). Upon sensing of pathogens, NLRs and AIM2/IFI16 trigger the formation of the inflammasome, a cytosolic molecular platform that recruits and activates caspase-1, initiating a program of pore formation in the plasma membrane of activated cells, with consequent cell rupture and release of cytosolic contents (Martinon et al., 2002; Fink and Cookson, 2006). The activity of caspase-1 also mediates the activation and controlled secretion of pro-inflammatory cytokines such as IL-1β and IL-18 (Thornberry et al., 1992; Ghayur et al., 1997; Gu et al., 1997). Although pyroptosis and cytokine secretion are both dependent on caspase-1 and occur concomitantly, it is not confirmed that cytokine release is mediated by induction of cell death pathway. Recently, caspase-11 was shown to mediate a non-canonical pathway of inflammasome activation in response to Gram-negative bacteria, leading to pyroptosis and release of cytokines such as IL-1α independently of caspase-1 activation (Kayagaki et al., 2011; Broz et al., 2012; Aachoui et al., 2013; Case et al., 2013; Casson et al., 2013). It was recently demonstrated that intracellular sensing of lipid A motif of lipopolysaccharide (LPS) induces caspase-11-dependent pyroptosis and NLRP3-dependent caspase-1 activation, with subsequent secretion of IL-1β and IL-18 (Hagar et al., 2013; Kayagaki et al., 2013). Most strikingly, detrimental effects of exacerbated inflammation during systemic infectious are possibly mediated by caspase-11, but not caspase-1 (Kang et al., 2002; Kayagaki et al., 2011, 2013; Hagar et al., 2013). This novel caspase-11-mediated inflammasome may operate synergistically with the other caspase-1- mediated inflammasomes for the recognition of pathogenic bacteria encoding type III/type IV secretion systems or escaping the vacuole (Figure 1).

Activation of the inflammasome, with consequent induction of pyroptosis, has been demonstrated for several microbial pathogens (Osawa et al., 2011; Lamkanfi and Dixit, 2012; Lima-Junior et al., 2013; Silva et al., 2013). In the case of bacterial pathogens, pyroptosis is a mechanism that effectively contributes to infection control (Miao et al., 2010a, 2011; Terra et al., 2010). Evolutionary pressure thus, shaped modulation of the inflammasome activation, with consequent inhibition of pyroptosis, as a subversion strategy found among microbial pathogens. Bona-fide intracellular pathogens (such as bacteria and viruses that modulate host cell functions through secretion systems and secreted proteins) use diverse strategies to evade recognition and inflammasome activation. However, the molecular mechanisms of inflammasome inhibition by pathogens remain largely unknown. Herein, we review the current knowledge on the mechanisms of inflammasome and pyroptosis suppression by pathogenic bacteria. We discuss the importance of inflammasome subversion to their pathogenesis and highlight recent findings on the diverse strategies adopted by bacterial pathogens to inhibit the activation of the inflammasome and how they affect pyroptosis.

## Mechanisms of pyroptosis inhibition by bacterial pathogens

### *Yersinia* spp.

The pathogenic Gram-negative bacteria belonging to the genus *Yersinia* have a tropism to target lymphoid tissues, inducing distinct types of host cell death in the course of infection. The three human pathogens of the genus, *Y. pestis, Y. pseudotuberculosis*, and *Y. enterocolitica* share a virulence plasmid encoding a conserved type III secretion system (T3SS) and a few identified effector proteins known as Yops (*Yersinia* outer proteins: YopE, YopT, YopH, YopM, YopA/O, and YopJ/P) (Trosky et al., 2008). The injection of Yops into infected cells allows the modulation of several signaling pathways and immune responses by *Yersinia*, including cell death. However, as the control of *Yersinia* multiplication is affected by a complex interplay of distinct types of cell death in different types of infected cells, it is likely that the demise of infected cells not only contributes to pathogenesis but also signals to mount an effective immune response (Philip and Brodsky, 2012). For instance, the early stage of infection is characterized by induction of apoptotic-like death of macrophages and dendritic cells, and YopJ, YopP (the homologous of YopJ in *Y. enterocolitica*) and YopK have already been implicated in this process, with evidence that apoptosis contributes to bacterial persistence *in vivo* (Mills et al., 1997; Monack et al., 1997, 1998; Ruckdeschel et al., 1998, 2001; Grobner et al., 2007; Peters et al., 2013). Translocation of YopJ is also implicated in late proinflammatory lytic cell death, independently of caspase-1 (Lilo et al., 2008). Although the molecular mechanisms triggering the inflammasome and caspase-1 activation in response to *Yersinia* are largely unknown, recognition of YopJ leads to differential regulation of inflammasome responses. Secretion of IL-1β in response to translocated YopJ requires caspase-1, Nlrp3, and Asc adaptor, whereas caspase-1 activation occurs in the absence of Nlrp3, Nlrc4, and Asc (Brodsky et al., 2010; Zheng et al., 2011). Proinflammatory cell death of infected macrophages mediated by YopJ does not require other inflammasome components such as Nlrp3, Nlrc4, and Asc, corroborating the lack of inflammasome participation in this process (Brodsky et al., 2010; Zheng et al., 2011). Moreover, T3SS recognition also induces caspase-1 activation and IL-1β secretion, requiring the inflammasome adaptor Asc and mediated by both Nlrp3 and Nlrc4, possibly in synergy (Brodsky et al., 2010). Notably, recognition of *Yersinia* T3SS also triggers caspase-1 mediated pyroptosis, independently of the effector YopJ, Nlrp3, Asc, and Nlrc4 (Bergsbaken and Cookson, 2007; Brodsky et al., 2010). Diverse effector proteins secreted by T3SS of *Yersinia* has already been shown to negatively modulate inflammasome activation with associated impairment of pyroptosis in response to *Yersinia* recognition. This accumulating evidence corroborates that although cell death processes might play different roles in the pathogenesis of *Yersinia*, evasion of inflammasome activation and inflammatory burst caused by pyroptosis should be important to bacterial success.

Regulation of cytotoxicity by differential secretion of YopJ is one of the processes that impacts virulence of *Yersinia*. Mutants of *Y*. *pseudotuberculosis* lacking YopJ do not induce cell death but fail to disseminate, showing that YopJ is required for optimal virulence (Monack et al., 1998). On the other hand, secretion of reduced levels of YopJ contributes to the pathogenesis of *Yersinae in vivo*. In this sense, cytotoxicity of dendritic cells induced by *Yersinia* positively correlated with the level of secretion of YopJ/P, but enhanced cytotoxicity in response to infection with *Y. pseudotuberculosis* ectopically expressing highly secreted YopP reduced virulence *in vivo*, causing an attenuated infection of the oral mucosa (Brodsky and Medzhitov, 2008). However, recent data demonstrated that caspase-1 deficiency does not impair the control of infection by hypercytotoxic *Y. pseudotuberculosis* (ectopically expressing YopP) (Zheng et al., 2012). Thus, it is possible that regulation of the levels of YopJ secretion might not be a subversion strategy to downregulate the induction of pyroptosis mediated by YopJ-dependent activation of the inflammasome.

The effectors YopE and YopT, which inactivate Rho GTPases that regulate cytoskeleton rearrangements (Cdc42, Rac1, Rho), were also shown to inhibit secretion of IL-1β in macrophage-like cells infected with *Y. enterocolitica* (Schotte et al., 2004). YopE also reduced cytotoxicity in these cells. Although YopE and YopT reduce activation of overexpressed caspase-1 and inhibit cell death in response to caspase-1 overexpression, the precise mechanism of inhibition of the inflammasome in macrophages has not been examined in detail. In the case of *Y. pseudotuberculosis*, there is no evidence that YopE and YopT play a role in inflammasome modulation (Larock and Cookson, 2012).

The caspase-1 activation, secretion of IL-1β and pyroptosis mediated by inflammasome recognition of T3SS in infected macrophages was shown to be inhibited by the effector YopK (Brodsky et al., 2010). This protein is secreted into the host cell cytosol and interacts with the T3SS, possibly leading to inhibition of inflammasome activation by impairment of recognition of the bacterial T3SS translocon structure. Activation of the inflammasome by mutants of *Y. pseudotuberculosis* lacking YopK leads to bacterial clearance *in vivo*, indicating a role of inflammasome inhibition by YopK in bacterial pathogenesis, promoting pathogen multiplication and dissemination. However, whether virulence mediated by inhibition of the inflammasome by YopK requires regulation of pyroptosis is yet only suggestive.

A recent report revealed that *Yersinia* also directs inhibition of caspase-1 and consequent inhibition of pyroptosis in infected macrophages through aT3SS-dependent effector (Larock and Cookson, 2012). The effector YopM binds to the active site of caspase-1 through a four amino acid motif similar to the sequence of the caspase-1 substrate YVAD and poxvirus protein CrmA, thus, sequestering the molecule and abrogating its interaction with the molecular platform formed by Nlrp3 and Asc in infected macrophages. Inhibition of caspase-1 activation by YopM impaired induction of pyroptosis, demonstrating that the effector modulates inflammatory cell death during infection. Importantly, absence of YopM impaired virulence of *Y. pseudotuberculosis in vivo*, suggesting that inhibition of caspase-1-dependent cell death and cytokine secretion should play a role in the pathogenesis of *Yersinia.*

How regulation of different types of cell death by *Yersinia*, i.e., apoptosis, pyroptosis and possibly necrosis, determines the balance between promotion of effective immune responses and successful immunomodulation, dissemination, and growth of the pathogen is yet to be understood.

### Pseudomonas aeruginosa

The Gram-negative bacterium *P. aeruginosa* is an opportunistic extracellular pathogen ubiquitously found in the environment. Antibiotic-resistance and vast distribution make *P. aeruginosa* a major source of nosocomial acute infection of immunocompromised individuals and infection associated to the use of contaminated medical devices. *P. aeruginosa* is also often associated to the infection of chronic cystic fibrosis patients (Garau and Gomez, 2003). The bacteria express a functional T3SS through which four known effectors, exoenzyme S (ExoS), ExoT, ExoU, and ExoY are secreted into host cell (Engel and Balachandran, 2009). Of note, expression of the exoenzymes varies among the different strains of *P. aeruginosa* (Engel and Balachandran, 2009). Activity of these effectors trigger signaling cascades, such as synthesis of cAMP (ExoY), cleavage of phospholipids (ExoU), and modulation of cytoskeleton dynamics (ExoS, ExoT, ExoU) that potentially can lead to activation of cell death pathways, although cytotoxic effects have been described to ExoS and ExoU only (Pederson and Barbieri, 1998; Sato and Frank, 2004).

The contribution of the inflammasome to recognition of *P. aeruginosa* by macrophages has been extensively described. The Nlrc4 receptor plays a major role in activation of caspase-1 in macrophages infected with pathogenic bacteria. Activation of the Nlrc4 inflammasome is triggered upon recognition of bacterial flagellin and the T3SS secretion system (Franchi et al., 2007; Sutterwala et al., 2007; Galle et al., 2008; Miao et al., 2008). Recognition of the T3SS rod component also occurs, dependent on Nlrc4 in a process mediated by activation of theNaip2 protein (Miao et al., 2010b; Zhao et al., 2011). Moreover, a toxin encoded by the *rhsT* gene of *P. aeruginosa* induces inflammasome activation and cytotoxicity in response to the bacteria, contributing to bacterial clearance *in vivo* (Kung et al., 2012). Activation of Nlrc4 inflammasome in response to *P. aeruginosa* mediates pyroptotic cell death and IL-1β secretion. Both processes have been shown to contribute to control of infection *in vivo*, although recent data argues that neutrophils, instead of macrophages, are the main source of IL-1β in infected mouse. Moreover, the IL-1β production by neutrophils occurs independently of bacterial flagellin, Nlrc4 or caspase-1 (Karmakar et al., 2012; Cohen and Prince, 2013). Thus, the contribution of the inflammasome to control of infection by *P. aeruginosa* may be further investigated.

Importantly, the T3SS-dependent effector proteins of *P. aeruginosa* have been shown to inhibit the inflammasome activation in macrophages both *in vitro* and *in vivo.* From the four described effectors secreted by *P. aeruginosa* T3SS, ExoS, and ExoU inhibit inflammasome-dependent responses, arguing that if not inhibited this pathway can play a pivotal role in immune responses and bacterial clearance.

It has been shown that ExoS deficiency leads to the secretion of cleaved IL-1β in both alveolar macrophages and in the lungs of mice infected with *P. aeruginosa* (Galle et al., 2008). ExoS is a bifunctional protein containing an amino-terminal Rho GTPase Activating Protein (GAP) domain, which modifies host cell targets that control the cytoskeleton, such as Cdc42, Rho and Rac1; and a carboxy-terminal ADP-ribosyltransferase domain (ADPRT) with ribosylation activity causing cytoskeleton rearrangements. Of note, the ADPRT domain, but not the GAP domain of ExoS, is essential to inhibition of IL-1β mediated by the exoenzyme. The importance of ribosylation activity of ExoS in this process allows speculating that modulation of host cell cytoskeleton dynamics is a possible mechanism through which ExoS inhibits the inflammasome. For instance, it has been recently reported that organelle transportation activity of microtubules is critical for activation of the inflammasome mediated by Nlrp3 (Misawa et al., 2013). Finally, ExoS induces caspase-3 dependent apoptotic cell death in response to infection but pro-inflammatory death of macrophages is also increased in the infection of macrophages with mutants lacking ExoS. Whether this effect is due to a putative ExoS-mediated inhibition of caspase-1-dependent pyroptosis or caused by cytotoxic effects independent of inflammasome activation has yet to be addressed.

For ExoU, it has been demonstrated that this exoenzyme inhibits Nlrc4-dependent caspase-1 activation and IL-1β in a process dependent on its phospholipase A2 activity (Sutterwala et al., 2007). However, pyroptosis triggered by Nlrc4 and caspase-1 was not modulated by ExoU, which is suggestive that the cytotoxic effect induced by the exotoxin may be due to non-apoptotic, caspase-1-independent necrosis (Sutterwala et al., 2007).

In summary, whether pyroptosis is involved in the pathogenesis of *P. aeruginosa* or whether it contributes to an efficient immune response by the host is still uncertain. However, differential induction and modulation of specific cell death pathways by the exotoxins of the pathogen, as well as clear inhibition of specific responses of the inflammasome by them, make *P. aeruginosa* a valuable model to investigate the role of different cell death pathways to the outcome of host-pathogen interaction and thus, should be further explored.

### Vibrio parahaemolyticus

Pathogenicity of *V. parahaemolyticus*, a Gram-negative extracellular bacterium associated mostly with seafood-borne gastroenteritis, relies on the expression of two thermostable pore-forming hemolysins (TDHs, namely TdhA and TdhS) and two sets of chromosome-encoded T3SS (T3SS-1 and T3SS-2) (Makino et al., 2003). A recent report demonstrated that *V. parahaemolyticus* has been shown to induce robust activation of the inflammasome dependent on multiple mechanisms (Higa et al., 2013). *V. parahaemolyticus* TDHs activate Nlrp3-dependent inflammasome (mainly through recognition of TdhA) and bacterial T3SS-1 induces inflammasome activation mediated by both Nlrp3 and Nlrc4. In addition, bacterial flagellin triggers the Nlrc4 inflammasome. Notably, recognition of TDHs and T3SS-1 were required to induce caspase-1-dependent pyroptosis in response to infection. In addition, inflammatory cell death independent of caspase-1 was also observed, suggesting that other pathways may be involved in the induction of cell death in response to *V. parahaemolyticus*. Notably this same report described a regulatory role for inflammasome activation mediated by the T3SS effectors VopQ and VopS, encoded in the pathogenicity island *h1* of Chromosome I. VopQ and VopS inhibited activation of the Nlrc4-dependent inflammasome upon recognition of T3SS-1. VopQ and VopS synergize to inhibit cleavage of caspase-1 and secretion of cleaved IL-1β, but any effect on pyroptosis has yet to be determined. In fact, complete deletion of *h1* decreased pyroptosis in response to infection, suggesting that other effectors of the bacteria also encoded in the region *h1* may be important for specific induction of pyroptosis by *V. parahaemolyticus.*

The effector VopQ is also known to be a determinant to the induction of autophagy in HeLa cells infected with *V. parahaemolyticus* (Burdette et al., 2009). Higa et al. (2013) demonstrated that VopQ induced autophagy in murine macrophages in response to infection. In addition, suppression of autophagic pathway by knocking down of Atg5 impaired the inhibition of IL-1β secretion mediated by VopQ, supporting a possible role of autophagy in inflammasome inhibition by VopQ. Importantly, induction of autophagy has been previously shown to negatively regulate inflammasome activation dependent on Nlrp3 (Saitoh et al., 2008). How the induction of autophagy mediated by VopQ could possibly mediate inflammasome suppression by the effector remains elusive, but it is possible that Nlrc4-mediated inflammasome activation may also be regulated by autophagy.

In the case of VopS, it is observed that the effector binds and inactivates endogenous Cdc42, which could account as a mechanism for inhibition of Nlrc4-inflammasome (Higa et al., 2013). As mentioned above, inhibition of inflammasome responses by the effectors ExoS of *P. aeruginosa* and YopE of *Y. enterocolitica* relies on their GAP activity that mediates inactivation of Rho GTPases (Schotte et al., 2004; Galle et al., 2008). No molecular role for regulation of inflammasome activation by active Rho GTPases has been demonstrated yet, but evidence suggests that these molecules may participate in inflammasome activation in response to pathogens. For instance, the SP-1 effector SopE of *Salmonella enterica* serovar Typhimurium, an activator of Rho GTPases induces inflammasome activation by stromal cells in response to bacterial recognition (Muller et al., 2009). Importantly, activation of caspase-1 by SopE requires modulation of Rac1 and Cdc42 by the bacterial effector. Another recent report revealed that activation of Rac1 in response to infection is important to NLRP3/ASC-dependent caspase-1 activation in response to *Chlamydia pneumoniae* by human mononuclear cells (Eitel et al., 2012). Importantly, a recent work showed that type I IFN signaling inhibits Rac1, with consequent repression of Nlrp3 inflammasome in macrophages (Inoue et al., 2012). These evidences reinforce a putative role of signaling pathways controlled by Rho GTPases in the modulation of inflammasome activation in response to pathogens, possibly inducing pyroptosis. How the activity of Rho GTPases in inflammasome activation, as well as modulation as a subversion strategy targeting immune responses may thus, be further explored.

### Chlamydia trachomatis

The obligate intracellular Gram-negative pathogen *C. trachomatis* is the causative agent of infections of the conjunctiva and urogenital tract commonly evolving to severe complications such as blindness, pelvic inflammatory disease, ectopic pregnancy, and infertility. The bacteria rely on the expression of a T3SS and secretion of effector proteins to adhere, invade, and establish a replicative inclusion (parasitophorous vacuole) in the target cells (Valdivia, 2008; Betts et al., 2009). Besides the T3SS-dependent effectors protein, the chlamydial protease-like activity factor (CPAF) is pivotal in the molecular pathogenesis of *C. trachomatis*, modulating host responses and stability of bacterial inclusion (Paschen et al., 2008). CPAF is translocated through the general secretory pathway, eventually reaching the host cell cytosol (Zhong et al., 2001). This effector is suggested to regulate by cleavage at least 16 host targets, interfering with several processes such as: proapoptotic signaling (Zhong et al., 2001; Pirbhai et al., 2006); expression of antigen presentation molecules (MHC) (Zhong et al., 2001); organization of host cell cytoskeleton (Dong et al., 2004; Kumar and Valdivia, 2008); control of cell cycle (Paschen et al., 2008) and NF-κB signaling pathway (Christian et al., 2010). Although host substrates were demonstrated to be cleaved by CPAF in cell lysates, enzymatic activity of CPAF may not be necessarily required *in situ* to exert its regulatory functions on host cell proteins (Chen et al., 2012). Nevertheless, CPAF has also been implicated in modulation of chlamydial proteins. It has been demonstrated that CPAF cleaves chlamydial T3SS-dependent effectors in cell-free systems and in infected cells, with evidences that CPAF proteolytic activity toward *C. trachomatis* effectors prevents superinfection and coordinates the formation and the integrity of the inclusion-containing the bacteria (Jorgensen et al., 2011).

In epithelial cells, which are the primary sites of infection by *C. trachomatis*, as well as in human monocytes and dendritic cells, NLRP3 and ASC mediate inflammasome-dependent activation of caspase-1 and secretion of cytokines in response to *C. trachomatis* (Lu et al., 2000; Gervassi et al., 2004; Abdul-Sater et al., 2009, 2010). However, the role of the inflammasome in the control of the infection by the pathogen is still controversial. Asc- and caspase-1-deficient mouse fibroblasts are resistant to infection by *C. trachomatis* (Jorgensen et al., 2011). In addition, in a mouse model of infection with *C. muridarum*, wild-type and caspase-1 deficient mice equally controlled the replication of the bacteria *in vivo* (Cheng et al., 2008). Of note, caspase-1-deficient mice displayed reduced inflammatory damage in the urogenital tract, suggesting that inflammasome activation may contribute to the pathology of infection by *Chlamydia* (Cheng et al., 2008).

Still, *C. trachomatis* regulates caspase-1-dependent cell death through the activity of CPAF. The use of a specific inhibitor of CPAF, design to overcome the refraction of the bacteria to genetic manipulation, revealed that CPAF activity inhibits ASC and caspase-1-dependent cell death in the early times of infection of epithelial cells with *C. trachomatis.* Late activation of caspase-1 occurs in epithelial cells and pharmacological inhibition of caspase-1 reduces bacterial growth in these cells, corroborating the importance of regulation of inflammasome activation to the pathogenesis of *C. trachomatis* (Abdul-Sater et al., 2009). However, a role for pyroptosis for caspase-1-dependent susceptibility to infection is still speculative. The mechanism of inhibition of early pyroptotic cell death by CPAF is yet to be understood and it is not ruled out that the protease directly interferes with inflammasome formation. Still, one interesting possibility proposed by Jorgensen et al. (2011) is that CPAF may function as a metaeffector (Kubori et al., 2010), regulating the pool of T3SS effectors in the host cytosol by proteolysis and also avoiding the accumulation of putative PAMPs to be sensed by cytosolic NLRs, possibly providing a novel mechanism of pathogenic modulation of the inflammasome.

### Francisella tularensis

Tularemia, a life-threatening infectious disease of the respiratory tract, is caused by the Gram-negative intracellular pathogen *Francisella tularensis.* Inside infected macrophages, the main target of infection, *F. tularensis* escapes the vacuole and replicates within the cytosol. However, in contrast to most of the intracellular pathogens, the bacteria do not rely on the activity of exotoxins or encoded T3SS and T4SS secretion systems and related effectors to modulate host cell functions (Larsson et al., 2005), and virulence mechanisms of the pathogen remain largely unknown (Broms et al., 2010; Meibom and Charbit, 2010). The inflammasome plays a pivotal role in recognition and control of infection by *F. tularensis* in experimental models of infection. The bacteria trigger activation of Aim2/Asc-dependent inflammasome in mouse macrophages (Mariathasan et al., 2005; Fernandes-Alnemri et al., 2010; Jones et al., 2010; Rathinam et al., 2010). Recognition of the bacteria by this Aim2/Asc leads to activation of caspase-1, secretion of IL-1β and IL-18, pyroptosis and culminate in the control of bacterial replication in macrophages and *in vivo*. In addition, Aim2 and Asc were proposed to trigger caspase-1-independent, caspase-8, -9, -3-mediated apoptosis of macrophages in response to infection with *F. tularensis*, contributing to restriction of bacterial replication in these cells (Pierini et al., 2012). Finally, whereas Nlrp3 is dispensable for inflammasome activation in murine macrophages, NLRP3 and AIM2 are suggested to play a role in human monocytic cells (Mariathasan et al., 2006; Fernandes-Alnemri et al., 2010; Jones et al., 2010; Atianand et al., 2011).

Absence of Aim2 activation by *mviN* and *ripA* mutants of *F. tularensis* have been initially reported (Huang et al., 2010; Ulland et al., 2010), but the lack of these encoded factors was shown to compromise the integrity of bacteria and enhance intramacrophage lysis of mutant bacteria and release of DNA into the host cell cytosol (Peng et al., 2011). Besides mviN and ripA, mutations on core components of the type VI secretion system of *F. tularensis* also affect activation of the inflammasome (Barker et al., 2009; Broms et al., 2012), therefore, it is still possible that this lack of activation may be due to an inherited defect in phagosome escape of this bacteria.

Recent data, however, suggests that the bacteria actively repress inflammasome signaling by the effector protein encoded by *FTL_0325*, a process that may contribute to repression of IL-1β secretion and bacterial growth *in vivo* (Dotson et al., 2013). The authors observed that virulent *F. tularensis* subsp. *tularensis* and *holarctica* fail to induce a robust activation of the inflammasome in the early times of the infection in comparison to attenuated *F. tularensis* subsp. *novicida*. Mutations in *FTL_0325* gene of *F. tularensis* subsp. *holarctica* (live vaccine strain -LVS) do not alter bacterial fitness whilst it exacerbates the synthesis of pro-IL-1β. In addition, mutants lacking *FLT_0325* also induce higher levels of caspase-1 activation dependent on Aim2 and Tlr2 and secretion of IL-1β dependent on Tlr2, Aim2, and Nlrp3 in the early periods of infection. Importantly, suppression of Aim2-dependent inflammasome activation by FLT_0325 inhibits pyroptosis in response to infection by *F. tularensis* LVS in macrophages (Dotson et al., 2013). Whether pyroptosis repression contributes to pathogenesis *in vivo* is still unclear.

### Shigella flexneri

Bacillary dysentery in humans is caused by mucosal infection with the Gram-negative intracellular pathogen *S. flexneri*. The bacteria express a functional T3SS, through which sequential delivery of bacterial effectors into host cell cytosol promotes pathogenesis (Ogawa et al., 2008). In addition, recognition of *S. flexneri* PAMPs elicits immune responses that paradoxically contribute to bacterial success (Phalipon and Sansonetti, 2007). *Shigella* invades the epithelia through the M cells of the mucosa barrier, subsequently infecting resident macrophages and dendritic cells. Once within these cells, the bacteria lyse the vacuole, replicates in the host cell cytosol and eventually triggers inflammatory cell death. This inflammatory burst and consequent neutrophil recruitment promotes basolateral invasion and dissemination of *S. flexneri*, followed by their entry into epithelial cells, renewed replication of bacteria and further dissemination along the epithelia using a cell-to-cell spread mechanism. However, the infection of epithelial cells generates an early genotoxic stress that could potentially cause necrotic death and bacterial control; bacterial replication inside these cells, suggests that *Shigella* also antagonizes cell death. In this way, *S. flexneri* concerted modulation of pro-death and pro-survival signaling pathways potentially allow bacterial circulation among different host compartments, maintenance of a replicative niche and a mechanism to circumvent the innate immune response (Schroeder and Hilbi, 2008; Ashida et al., 2011).

Signaling through Nlrc4inflammasome pathway, mediated by recognition of the rod component MxiI of the T3SS apparatus culminates in caspase-1 activation, pyroptosis and IL-1β and IL-18 secretion (Suzuki et al., 2007; Miao et al., 2010b). The T3SS effector protein IpaB also induces pyroptosis and IL-1β dependent on caspase-1 (Chen et al., 1996; Hilbi et al., 1998). Although physical interaction of caspase-1 and IpaB has been demonstrated, recent data support a mechanism of ion channel formation by oligomerization of IpaB in the host cell membrane, possibly inducing Nlrc4 and Asc-dependent inflammasome activation that culminates into caspase-1 activation and pyroptosis (Senerovic et al., 2012). Inflammasome activation in infected macrophages, caspase-1 activation, pyroptosis and secretion of inflammatory cytokines can correspond to the inflammatory burst associated to shigellosis. Importantly, inflammatory burst induced by *S. flexneri*, associated with bacterial invasion and dissemination as well as resolution of infection by a competent host, requires caspase-1, IL-1β, and IL-18 (Sansonetti et al., 1995, 2000). It is likely that fine modulation by *Shigella* of pyroptosis in infected macrophages could favor bacterial basolateral dissemination but avoid the potential restriction of infection associated with robust immune signaling. Nonetheless, a specific molecular mechanism underlying this putative process has not yet been revealed.

In the case of nonmyleoid epithelial cells, acute infection by *Shigella* induces necrotic cell death pathways as a consequence of mitochondrial damage as well as due to genotoxic stress through activation of calpain. The activation of calpain is a process mediated by the bacterial effector VirA with complex consequences, promoting bacterial uptake, inhibition of early pro-apoptotic signaling by degradation of p53 but also induction of late necrosis that contributes to invasion (Bergounioux et al., 2012). However, death of infected cells is supposed to be modulated by the bacteria to support intracellular bacterial growth inside epithelial cells thus, favoring primary tissue colonization. In this sense, activation of pro-survival NF-κB signaling pathway through recognition of bacterial PAMPs by Nod1 and Rip2 possibly counterbalances necrotic cell death (Carneiro et al., 2009).

A recent report revealed that mutants of *S. flexneri* lacking the expression of the T3SS effector protein OspC3 induce early pyroptotic cell death upon infection by *S. flexneri* of human epithelial cell lines (Kobayashi et al., 2013). In agreement, *ΔospC3 S. flexneri* increases mucosal cell death and inflammatory infiltrate in the intestine of infected guinea pigs, with associated reduction of bacterial growth in the epithelia without affecting bacterial invasiveness. Of note, pyroptosis induced by *ΔospC3 S. flexneri* specifically requires caspase-4, the human homolog of murine caspase-11, but not caspase-1. OspC3 reduces catalytic activity of caspase-4, also decreasing cell death induced by overexpression of p19 catalytic subunit of casp-4. Caspase-4 physically interacts with OspC3 through the catalytic site in the p19 subunit of the active caspase-4. Interaction and inhibition of pyroptosis induced by caspase-4 also requires the C-terminal Ankyrin repeat-containing domain of OspC3 (ANK), a eukaryotic-like domain predicted to mediate protein-protein interactions. Most strikingly, certain motifs in the ANK domain of OspC3 share high similarity to other bacterial and viral proteins, including those encoded by *Legionella pneumophila, Coxiella burnetii, Rickettsia rickettsia*, and vaccinia virus. This first demonstration of a pathogen effector protein that inhibits non-canonical induction of pyroptosis is suggestive that this mechanism might be a common strategy to modulate the induction of inflammatory responses among diverse pathogens. In the case of *Shigella*, it is likely that inhibition of caspase-4-dependent pyroptosis provides both the maintenance of epithelial replicative niche as well as evasion of early immune signaling.

### Legionella pneumophila

*L. pneumophila* is a Gram-negative intracellular bacterial pathogen that accidentally infects humans, causing a pneumonia-like disease in immunocompromised individuals. The pathogen resides within a cytosolic endosomal replicative vacuole (LCV), avoiding fusion with lysosomal vesicles and modulating diverse host cell functions to maintain the replicative niche. To this end, *L. pneumophila* secretes through a type IVB secretion system called Dot/Icm (**D**efective **o**rganelle **t**rafficking/**I**ntra**c**ellular **m**ultiplication) more than 300 effectors proteins into the host cell cytosol, which are mostly involved in the maintenance of the LCV and bear wide function redundancy (Hubber and Roy, 2010).

*L. pneumophila* is known to induce robust activation of the inflammasome by triggering different pathways. Bacterial flagellin secreted through the Dot/Icm system into host cell cytosol is recognized by the Naip5-Nlrc4-caspase-1 axis, triggering pyroptosis and Asc-dependent secretion of IL-1β (Amer et al., 2006; Molofsky et al., 2006; Ren et al., 2006; Zamboni et al., 2006; Lightfield et al., 2008; Case et al., 2009; Silveira and Zamboni, 2010). Of note, flagellin recognition via Naip5/Nlrc4/caspase-1 account to infection control *in vitro* and *in vivo* (Amer et al., 2006; Molofsky et al., 2006; Ren et al., 2006; Zamboni et al., 2006; Coers et al., 2007; Pereira et al., 2011). Moreover, data suggests that activation of caspase-7 dependent on this inflammasome pathway leads to LCV acidification and fast macrophage death, contributing to bacteria control *in vitro* (Akhter et al., 2009). In addition, Dot/Icm products induce flagellin-independent inflammasome activation regulated by caspase-11. Caspase-11 mediates macrophage pyroptosis and secretion of IL-1α, besides regulating Nlrp3/Asc-dependent secretion of IL-1β (Case et al., 2013). Importantly, evidence suggests that inflammasome-dependent pyroptosis and neutrophil recruitment mediate by IL-1β and IL-1α are important to bacterial clearance (Casson et al., 2013). Recently, secretion of IL-1α independent of caspase-1 and caspase-11 has also been shown to participate in neutrophil recruitment and infection control (Barry et al., 2013).

In contrast to the current knowledge on inflammasome activation by *L. pneumophila*, little is known about mechanisms of inflammasome subversion by the pathogen. Of note, *L. pneumophila* evolved cycling through different unicellular amebae protozoa in freshwater reservoirs, possibly conserving features that allow a broad host-range pathogen instead of those specific to provide resilience in specialized phagocytes (Ensminger et al., 2012). In this sense, the course of pathogen adaptation to adequate host, *L. pneumophila* may have encountered little selective pressure to evade PRRs recognition and immune responses (Massis and Zamboni, 2011). Consequently, it is possible that a reduced number of *L. pneumophila* effectors should be involved in subversion of innate immune responses of host macrophages such as inflammasome activation and pyroptosis, favoring the conservation of tools to hijack vesicles and organelles necessary to constant remodeling of the LCV. On the other hand, the recent description of a putative primitive immune-like system encoded in the genome of *Acanthamoeba castellanii* (Clarke et al., 2013) raises the possibility of existence of environmental pressure that could have favored natural selection of bacteria provided with evasion mechanisms against host immune response.

Of note, upregulation of non-apoptotic genes by activation of NF-κB in a Dot/Icm-dependent manner counterbalance the activation of caspase-3-mediated apoptotic pathway upon infection, indicating that *L. pneumophila* can modulate host cell death (Abu-Zant et al., 2005, 2007). Early activation of caspase-3 is induced by multiple bacterial secreted effectors and plays a role in the arrested maturation of nascent bacteria-containing phagosome through the endocytic pathway (Gao and Abu Kwaik, 1999; Zink et al., 2002; Molmeret et al., 2004; Zhu et al., 2013). Importantly, caspase-3 activation accounts for restriction of bacterial replication in dendritic cells (Nogueira et al., 2009). Although direct inhibition of the inflammasome and pyroptosis by *L. pneumophila* effector proteins has not yet been demonstrated, the Dot/Icm effector SdhA is required for bacterial replication in macrophages (Laguna et al., 2006). SdhA is important to avoid Aim2-dependent inflammasome activation in response to recognition of *L. pneumophila* DNA (Ge et al., 2012). Macrophages infected with mutants lacking *sdhA* gene trigger Aim2-dependent activation of caspase-1, secretion of IL-1β and pyroptosis, which is reversible by infection with genetically complemented bacteria. However, as in the case of *F. tularensis* and the genes *mviN* and *ripA*, it will be important to determine whether the activation of Aim2 inflammasome in response to infection with *sdhA* mutants is not an indirect effect of bacterial DNA release in the cytosol as a consequence of compromised integrity of LCV and bacterial degradation. Interestingly, a recent report suggested that induction of autophagosome turnover dependent on recognition of virulent flagellate *L. pneumophila* through Naip5/Nlrc4/pro-caspase-1 regulates pyroptosis triggered by the same pathway (Byrne et al., 2013). However, the mechanism by which inflammasome components promote the autophagic flux and how the induction of autophagy regulates pyroptosis remain elusive. Paradoxically, *L. pneumophila* inhibits autophagy through irreversible inactivation of Atg8 mediated by the effector RavZ (Choy et al., 2012). Whether autophagy contributes to infection or boosts immunity in response to *L. pneumophila* should be further explored.

### Coxiella burnetii

Similarly to *Legionella pneumophila*, the Gram-negative, obligate intracellular bacteria and human pathogen *Coxiella burnetii* express the unique type IVB Dot/Icm secretion system (McDonough et al., 2012). Although the two pathogens are also closely related in phylogenetic analysis, *C. burnetii* is a bona-fide mammalian pathogen, with a strong tropism for alveolar macrophages in infected humans. Their distinct natural history is evident in the strikingly divergent life style adopted by the bacteria once inside the host cells. *C. burnetii* demands an acidified environment for morphological development, Dot/Icm expression and replication, which is accomplished by active maturation of bacteria-containing vacuole through the endosomal pathway, culminating in fusion with recruited lysosomal vesicles and formation of a large replicative vacuoles (LRV) (Newton and Roy, 2011). Of note, *C. burnetii* is capable of modulating several cellular processes to both remodel the LRV as well as to escape bacterial recognition and control. The genome of *C. burnetii* encodes more than 200 putative candidates for Dot/Icm secretion, of those roughly 25% have been shown to be effectively expressed and secreted in to host cell cytosol, with just a few with a demonstrated functionality (Van Schaik et al., 2013). It is possible that functional redundancy among *C. burnetii* effectors is reduced in comparison to *L. pneumophila*, as diverse genes have already been shown to affect LRV formation (Weber et al., 2013).

The mechanism of recognition and immune response to *C. burnetii* in macrophages remains elusive. Variations in the O-antigen of *C. burnetii* LPS are determinant to the virulence of the bacteria, with avirulent organisms expressing a truncated form of O-antigen in the LPS structure. Besides, differences in antigenic reactivity of *C. burnetii* LPS is determined by variation in the chemical composition of the O-polysaccharide chain (Narasaki and Toman, 2012). Although the bacterial lipopeptides are recognized by Tlr2, as demonstrated with infections performed purified molecules and with avirulent phase II *C. burnetii* (Zamboni et al., 2004), the virulent phase I bacteria avoid Tlr2 recognition by forming a protective structure that avoids exhibition of components of the bacteria cell wall for Tlr2 recognition (Shannon et al., 2005). In addition, the structure of the lipid A of the LPS of *C. burnetii* was also revealed and it was shown that lipid A derived from both virulent and avirulent bacteria antagonizes Tlr4 activation (Zamboni et al., 2004). Of note, antagonic engagement of Tlr4 by *C. burnetii* LPS is possibly a complex process. A recent report showed that virulent bacteria and their LPS trigger an impaired activation of the MAPK pathway in macrophages, which is important to avoid conversion of phagolysosomes hosting bacteria into degradative compartments containing cathepsin D (Barry et al., 2012).

The bacterium is also known to induce pro-survival pathways that sustain bacterial growth. Phase I and phase II *C. burnetii* induce sustained phosphorylation of anti-apoptotic host proteins Akt and Erk1/2 (Voth and Heinzen, 2009). Interaction of Beclin-1, a protein of autophagy, with anti-apoptotic Bcl2 in the membrane of the bacterial LRV prevents apoptosis of cells infected with *C. burnetii* (Vazquez and Colombo, 2010). In addition, *C. burnetii* inhibits caspase-3- dependent intrinsic pathway of apoptosis (Luhrmann and Roy, 2007; Voth et al., 2007), and the Dot/Icm effectors AnkG, CaeA, and CaeB have already been implicated in this process by distinguished mechanisms. The effector AnkG inhibits host cell apoptosis dependent on the interaction with p32, a host cytoplasmic protein implicated in pathogen-induced apoptosis (Luhrmann et al., 2010). Whereas a mechanism for inhibition of apoptosis by CaeA has not yet been demonstrated, the effector CaeB co-localizes with the mitochondria and its overexpression reduces the loss of MOMP (**m**itochondria **o**uter **m**embrane **p**ermeabilization) induced by activation of the apoptosis pathway (Klingenbeck et al., 2012).

A role of NLRs and inflammasome activation in the recognition and control of *C. burnetii* infection has not been demonstrated so far, even though the bacteria is a bona-fide intracellular pathogen that express a functional secretion system, a hallmark for bacterial sensing by macrophages. Still, the capacity of the bacteria to thrive inside the macrophages throughout a slow replicative life cycle suggests that the bacteria might subvert inflammatory responses including the activation of the inflammasome. Future investigations should shed light in a possible role of the inflammasomes in host response to *C. burnetii*, as well as reveal novel mechanisms of bacterial subversion of the inflammasome and pyroptosis.

## Concluding remarks

**Figure 2:**
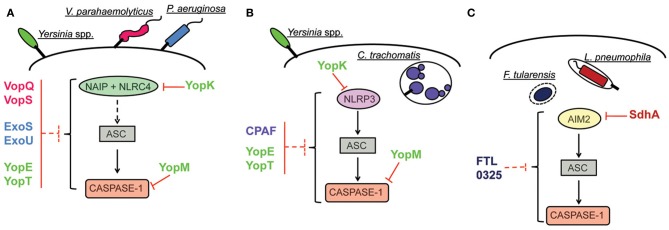
**Inhibition of inflammasome activation pathways by pathogenic bacteria. (A)** Inhibition of NAIP/NLRC4 inflammasome. The effector YopK of *Yersinia* is secreted into host cell cytosol by the T3SS and interacts with the translocon structure in the host cell cytosol interface; this interaction possibly prevents recognition by cellular receptors. The effectors VopQ/S of *V. parahaemolyticus* and ExoU of *P. aeruginosa* inhibit the NAIP/NLRC4 inflammasome by unknown mechanisms. In the case of the effectors YopE/T of *Yersinia* and ExoS of *P. aeruginosa*, interaction of the bacterial proteins with caspase-1 *in vitro* are suggestive of a putative mechanism for inflammasome inhibition by direct interaction with caspase-1. Inhibition of inflammasome activation by interaction with caspase-1 has been characterized for the effector YopM of *Yersinia*. **(B)** Inhibition of NLRP3 inflammasome. Inhibition of NLRP3-dependent inflammasome by YopK occurs as explained in **(A)**. The protein CPAF of *C. trachomatis* is a protease that can target bacterial effectors secreted into host cell cytosol and inhibits ASC-dependent inflammasome activation (that could be triggered by NLRP3) by unknown mechanisms. In the case of YopE/T, suggestion of inflammasome inhibition by direct interaction with caspase-1 indicates possible subversion of both NLRP3 and NAIP/NLRC4 activation pathways. In the case of YopM, demonstration of caspase-1 inhibition by direct interaction also suggests that both pathways can be subverted. **(C)** Inhibition of AIM2 inflammasome. The effector SdhA of *L. pneumophila*, required for bacterial growth, prevents bacterial DNA release into host cell cytosol, thus, avoiding recognition by host DNA receptor AIM2. In the case of *F. tularensis*, the protein encoded by bacterial gene *FTL_0325* also inhibits AIM2 inflammasome activation by unknown mechanisms.

Activation of the inflammasome is a broad host response that effectively contributes to innate immune response and infection control of a remarkably high number of infectious agents. Activation of this platform leads to inflammasome-dependent secretion of cytokines, induction of pyroptosis and restriction of pathogen replication, by mechanisms that are still obscure. As reviewed here, targeting inflammasome activation is a common evasion strategy of different species of bacterial pathogens. Importantly, different steps of the signaling cascade that leads to inflammasome activation are targeted by bacterial proteins. However, in most cases, the molecular mechanisms underlying inflammasome inhibition are still not fully understood. Few reports identified a direct interaction with the inflammasome effector molecule caspase-1, whereas others provided evidence of an interference with upstream signaling pathway (Figure 2, Table 1). Moreover, in some cases the inhibition of the inflammasome was verified in the level of caspase-1 activation and IL-1β secretion, without appropriate assessment of inhibition of pyroptosis. In this scenario, it should be considered that secretion of IL-1β and pyroptosis can be differentially regulated, with caspase-11 emerging as a master regulator of these processes. The contribution of caspase-11 in inflammasome activation in response to pathogens is possibly underscored because published literature on the activation of caspase-1 has been widely assessed using C57BL/6 mice double knockout for both caspase-1 and caspase-11 (Kayagaki et al., 2011). In this scenario, it will be important to reevaluate if the reported suppression of the inflammasome by bacterial proteins reviewed herein occur via inhibition of the canonical (caspase-1-dependent, caspase-11- independent) or non-canonical (caspase-11-dependent only) inflammasome. Finally, it is important to emphasize that dysfunctions on inflammasome signaling is intrinsically connected to the onset of diverse chronic inflammatory metabolically and autoimmune syndromes. Understanding the molecular mechanisms of pathogen subversion strategies for suppression of the inflammasome activation and elucidate how they specifically affect inflammasome responses will be critical to a comprehensive understanding of the bacterial pathogenesis and host response. Importantly, it may provide clues for the advance in the development of effective therapeutics to uncontrolled inflammation associated to systemic infections and chronic inflammatory diseases.

**Table 1:** **Summary of bacterial effectors that suppress inflammasome activation and their role on suppression of pyroptosis, as discussed in the main text**.

	**Bacterial effector**	**Mechanism of inflammasome inhibition**	**Inflammasome target**	**Effect on macrophage pyroptosis**	**References**
*Yersinia* spp.	YopK	Interaction with YopK possibly prevents recognition of T3SS	Metaeffector	Inhibition	Brodsky et al., 2010
	YopE (*Y. enterocolitica*)	Unknown, YopE interacts with caspase-1 *in vitro*	Unknown	Inhibition	Schotte et al., 2004
	YopM	Direct inhibition of caspase-1 activation	Caspase-1	Inhibition	Larock and Cookson, 2012
	YopT (*Y. enterocolitica*)	Unknown, YopT interacts with caspase-1 *in vitro*	Unknown	Not-described	Schotte et al., 2004
*P. aeruginosa*	ExoS	Unknown, dependent on ribosylation activity of ExoS	Unknown	Not-described	Galle et al., 2008
	ExoU	Unknown, dependent on phospholipase activity of ExoU	Unknown	No inhibition	Sutterwala et al., 2007
*V. parahaemolyticus*	VopQ	Unknown, dependent on host cell autophagy	Unknown	Not described	Higa et al., 2013
	VopS	Unknown, dependent on inhibition of Rho GTPase byVopS	Unknown	Not described	Higa et al., 2013
*C. trachomatis*	CPAF	Unknown, requires proteolytic activity of CPAF	Unknown	Inhibition	Jorgensen et al., 2011
*F. tularensis*	FTL_0325	Unknown	Unknown	Inhibition	Dotson et al., 2013
*S. flexneri*	OspC3	Direct inhibition of caspase-4	Caspase-4	Inhibition	Kobayashi et al., 2013
*L. pneumophila*	SdhA	Inhibition of bacterial DNA release	AIM2	Inhibition	Ge et al., 2012

### Conflict of interest statement

The authors declare that the research was conducted in the absence of any commercial or financial relationships that could be construed as a potential conflict of interest.
